# Ribose-Binding Protein Mutants With Improved Interaction Towards the Non-natural Ligand 1,3-Cyclohexanediol

**DOI:** 10.3389/fbioe.2021.705534

**Published:** 2021-07-23

**Authors:** Diogo Tavares, Jan Roelof van der Meer

**Affiliations:** Department of Fundamental Microbiology, University of Lausanne, Lausanne, Switzerland

**Keywords:** biosensor, periplasmic binding proteins, *Escherichia coli*, directed mutagenesis, fluorescence assisted cell sorting

## Abstract

Bioreporters consist of genetically modified living organisms that respond to the presence of target chemical compounds by production of an easily measurable signal. The central element in a bioreporter is a sensory protein or aptamer, which, upon ligand binding, modifies expression of the reporter signal protein. A variety of naturally occurring or modified versions of sensory elements has been exploited, but it has proven to be challenging to generate elements that recognize non-natural ligands. Bacterial periplasmic binding proteins have been proposed as a general scaffold to design receptor proteins for non-natural ligands, but despite various efforts, with only limited success. Here, we show how combinations of randomized mutagenesis and reporter screening improved the performance of a set of mutants in the ribose binding protein (RbsB) of *Escherichia coli*, which had been designed based on computational simulations to bind the non-natural ligand 1,3-cyclohexanediol (13CHD). Randomized mutant libraries were constructed that used the initially designed mutants as scaffolds, which were cloned in an appropriate *E. coli* bioreporter system and screened for improved induction of the GFPmut2 reporter fluorescence in presence of 1,3-cyclohexanediol. Multiple rounds of library screening, sorting, renewed mutagenesis and screening resulted in 4.5-fold improvement of the response to 1,3-cyclohexanediol and a lower detection limit of 0.25 mM. All observed mutations except one were located outside the direct ligand-binding pocket, suggesting they were compensatory and helping protein folding or functional behavior other than interaction with the ligand. Our results thus demonstrate that combinations of ligand-binding-pocket redesign and randomized mutagenesis can indeed lead to the selection and recovery of periplasmic-binding protein mutants with non-natural compound recognition. However, current lack of understanding of the intermolecular movement and ligand-binding in periplasmic binding proteins such as RbsB are limiting the rational production of further and better sensory mutants.

## Introduction

Periplasmic binding proteins (PBPs) form a family of proteins with a conserved bilobal structure (Berntsson et al., 2010; Chu Byron and Vogel, 2011). PBPs scavenge molecules (ligands) for the cell, which upon binding are presented to transport channels and/or to membrane receptors involved in chemotaxis (Binnie et al., 1992). Crystal structures and nuclear-magnetic resonance data have shown that PBPs can adapt two semi-stable conformations. In absence of the ligand most of the protein molecules adopt an open conformation, in which the binding site is exposed. In presence of the ligand, the molecule is buried in the binding pocket and the PBP adopts a closed conformation (Björkman and Mowbray, 1998; Li et al., 2013). This particular opening-and-closing and the fact that they locate in the periplasmic space make PBPs a potentially attractive protein class for biosensing purposes (Edwards, 2021). PBPs can be integrated in an *in vivo* hybrid signaling chain leading to expression of a reporter protein, which can be easily measured (Medintz and Deschamps, 2006; van der Meer and Belkin, 2010). In the context of this work, we focused on the ribose binding protein (RbsB) of *Escherichia coli*, which in presence of the natural ligand ribose changes from open to closed conformation. RbsB and its affinity for ribose have been deployed as a bioreporter system through use of a hybrid membrane receptor named Trz1 (Reimer et al., 2014). Trz1 consists of a fusion between the C-terminal part of the *E. coli* cytoplasmic EnvZ osmoregulation histidine kinase and the N-terminal periplasmic and membrane-spanning part of the *E*. *coli* Trg chemotaxis receptor (Baumgartner et al., 1994). Ribose-bound RbsB triggers the Trz1 autophosphorylation cascade, leading to OmpR phosphorylation and increasing its affinity for the *ompC* promoter. This yields increased transcription of reporter genes fused to P*_*ompC*_*.

Periplasmic binding proteins have been proposed as a flexible platform to design new ligand-binding pockets based on protein engineering approaches (Dwyer and Hellinga, 2004), with, however, very limited and controversial successes. Several studies attempted to engineer PBPs based on rational design and computational approaches, but this led to poorly foldable proteins (Boas and Harbury, 2008; Scheib et al., 2014; Banda-Vazquez et al., 2018). Other studies exploited natural properties of the PBPs to reduce or increase binding specificity (Amiss et al., 2007; Ko et al., 2017) or to graft binding-pockets between closely related PBPs (Scheib et al., 2014; Banda-Vazquez et al., 2018). We previously described six novel RbsB mutants with loss of binding to ribose and moderate but significant response to 1,3-cyclohexanediol (13CHD). These mutants were obtained through a combination of computational prediction of binding pocket mutations in RbsB and screening for gain of GFPmut2 fluorescence output in an *E. coli* bioreporter system (Tavares et al., 2019). However, all mutant proteins showed signs of poor stability, mis- or unfolding and potentially translocation problems compared to wild-type RbsB, suggesting that the poor inducibility by 13CHD may partly be due to protein instability caused by the introduced binding pocket substitutions. Indeed, other studies have shown that mutations both in the ribose binding pocket and protein periphery can destabilize the protein (Antunes et al., 2011; Reimer et al., 2017). This was refined by alanine replacement mutagenesis, in order to understand individual residue importance for RbsB folding and functioning (Vercillo et al., 2007; Reimer et al., 2017).

The goal of this work was to understand whether primitive binding of 13CHD by designed RbsB mutants (Tavares et al., 2019) can be improved by both rational and directed evolution approaches. We started with six mutant *rbsB* templates that were previously obtained (Tavares et al., 2019), which were used for random or site-directed mutagenesis, cloned into the *E. coli* GFPmut2 bioreporter strain and extensively screened by fluorescence-assisted cell sorting for improved GFPmut2 induction in presence of 13CHD. Potential gain-of-function mutants were separated and used for new rounds of mutagenesis and screening. Our hypothesis was that mutations in parts of RbsB outside the direct binding pocket may compensate folding defects and could lead to better functional PBP variants. Given that computational predictions on PBP folding are not sufficiently accurate yet, this procedure might open a route to profit from *de novo* computational binding pocket predictions to create primitive binding capacity and optimize protein functioning using random mutagenesis and selection.

## Results

### Random Mutagenesis of RbsB Protein Variants With Primitive 13CHD Affinity

We previously selected six RbsB mutants (named DT001, DT002, DT011, DT013, DT015, and DT016) that had lost the capacity to bind ribose, and instead had gained primitive affinity to 13CHD as new ligand (Tavares et al., 2019). Purified mutant proteins, however, displayed severe misfolding, poor stability and poor translocation into the periplasmic space (Tavares et al., 2019). In order to potentially improve mutant protein functionality, we used the respective *rbsB-DT* mutant genes as individual templates to produce random mutagenesis libraries (RML) using error prone PCR (ep-PCR). RMLs produced from each starting RbsB-variant were transformed into the *E. coli* bioreporter strain carrying the Trz1-*ompR-ompC’:gfpmut2* signaling chain (Reimer et al., 2014). Individual clones were encapsulated in alginate beads and grown to microcolonies, which were incubated with 1 mM 13CHD to induce GFP formation (Tavares et al., 2019).

Some 10 million beads, covering three times the estimated sizes of the RML002 and RML016 (derivatives of DT002 and DT016, respectively) were screened by fluorescence-activated cell sorting (FACS), separating beads with an 13CHD-induced fluorescence above the 98th percentile. 10^5^ beads were recovered, from which plasmid DNA was isolated and used as template for a new round of random mutagenesis. The new libraries (with estimated sizes of 5.5 × 10^6^ and 8.5 × 10^6^ clones) were again encapsulated, induced and screened, but now restricting recovery to the top 0.1% of GFPmut2 fluorescence. 6 × 10^3^ beads were collected, purified to individual clones, and screened in eight replicates in 96-well plates for 13CHD induction. This resulted in finding three mutants (named: DT020, DT021, and DT022) with consistent and up to 2.1-fold 13CHD induction, a significant increase and/or reduction of fluorescence background when compared with parental DT002 and DT016 (Table 1). Sequencing revealed a single different amino acid substitution in each of the three mutants (Table 1). Mutant DT020 had the exact same 1.5-fold induction as its parent DT016, but showed a 30% reduced fluorescence background intensity (*p* = 2.25 × 10^–5^, *n* = 12 replicates, Table 1). Mutants DT021 and DT022 displayed a small increase in fold induction to 1.66 ± 0.09 (*n* = 13 replicates) and 2.09 ± 0.16 (*n* = 14 replicates), respectively (Table 1).

**TABLE 1:** GPFmut2 fluorescence in *Escherichia coli* expressing wild-type- and mutant-RbsB proteins under uninduced and 13CHD-induced conditions.

**Wild-type or Variant**	**Parental Protein**	**GFPmut2 uninduced fluorescence^a^**	**Fold induction^b^ 1 mM 1,3-cyclohexanediol**	**Additional mutation(s)^c^**
RbsB	–	**25,226 ± 4,066^d^**	**0.91 ± 0.05**	–
DT016	RbsB	127,887 ± 12,650	1.51 ± 0.02 (Tavares et al., 2019)	**^e^**
DT020	DT016	**87,877 ± 21,152**	1.48 ± 0.07	V10I**^f^**
DT021	DT016	**151,009 ± 22,735**	**1.66 ± 0.09**	K206R
DT022	DT016	132,129 ± 25,700	**2.09 ± 0.16**	G89V
DT032	DT022	**95492 ± 34689**	**2.97 ± 0.37**	L170S
DT033	DT022	100,256 ± 50,431	**2.63 ± 0.59**	L201P S207P K250R
DT035	DT022	**95,073 ± 23,566**	**2.60 ± 0.50**	K5N
DT038	DT022	**49,432 ± 13,382**	**3.19 ± 0.48^g^**	L201V

*^*a*^Mean (±one *SD*) GFPmut2 fluorescence values of uninduced cultures (*n* = 8–14 replicates).*

*^*b*^Mean fold induction ± one *SD*, as the ratio of mean GFPmut2 fluorescence of induced cultures with 1 mM 1,3-cyclohexanediol, by that of uninduced cultures.*

*^*c*^Additional mutations in comparison to the parental protein.*

*^*d*^Values in bold indicate statistically significant fluorescence background and/or fold induction compared to DT016 (*p* < 0.05, *t*-test, equal variance).*

*^*e*^DT016 carries eight mutations compared to wild-type RbsB: F16S, N64V, D89V, R90S, T134A, F164W, F214A, and Q235M.*

*^*f*^Underlined substitutions are located on the 25 amino acids long signal peptide.*

*^*g*^Improvement compared to DT016 is the net gain in fold-induction, thus (3.19–1)/(1.51–1) = 4.38.*

Separate RMLs produced from the initial variants DT001, DT011, DT013, and DT015 (named RML001, RML011, RML013, and RML015, and with library sizes of 3.4 × 10^6^, 2.2 × 10^6^, 1 × 10^6^, and 4.1 × 10^6^ clones, respectively), were similarly encapsulated, induced with 13CHD and screened on an estimated three times library coverage for higher GFPmut2 fluorescence compared to non-induced conditions. In total, 151 beads were recovered that showed GFPmut2 fluorescence higher than any bead observed in non-induced conditions, which were purified and individually tested for 13CHD inducibility. Unfortunately, all mutants also showed significant increase in fluorescence in absence of 13CHD and none had induction levels above 1.5 times. Sequencing of some of these mutants showed gene deletions resulting in truncated RbsB mutant proteins. Pooled DNA from those 151 mutants used as template for a new library (estimated size of 1.5 × 10^6^ variants) did not yield further improvements. In contrast, almost all tested clones showed deletions of the *rbsB* variant open reading frames resulting in truncated proteins. These RMLs were therefore not further investigated.

### Random Mutagenesis of 2nd Generation Mutant RbsB Proteins With 13CHD Affinity

Because of the accumulation of truncated gene variants in the libraries we decided to create three new RMLs based on the newly isolated improved DT variants (DT020, DT021, and DT022, Table 1). These libraries (RML020, RML021, and RML022, with estimated sizes of 1.5 × 10^6^, 3 × 10^6^, and 2.5 × 10^6^ clones, respectively) were again encapsulated to individual cells, grown to microcolonies and screened both under uninduced and 13CHD-induced conditions. In this screening, only beads with a fluorescence signal higher than the maximum observed signal under non-induced conditions for the same number of screened beads, were collected. Four mutants were recovered, with consistent and significant increase of 13CHD-dependent induction of GFPmut2 fluorescence and/or reduced background in absence of 13CHD (Table 1, *n* = 8–14 replicates). Three of those mutants displayed a single amino acid substitution, and one (DT033) showed three substitutions (Table 1). In one case (DT035) the substitution affected an amino acid in the signal peptide. Mutants DT033 and DT035 showed a similar fold induction, around 2.6 times, in presence of 13CHD (*n* = 9–12 replicates, Table 1). Mutants DT032 and DT038 were the most promising, with fold-inductions of 2.97 ± 0.37 and 3.19 ± 0.48 times (*n* = 13–14 replicates, Table 1). This represents a 4.5-fold increase in induction compared to the parental mutant DT016 (Table 1). All four mutants displayed a reduction in GFPmut2 fluorescence in absence of inducer in comparison to parental DT016, except DT033. The highest reduction was observed with DT038, with a background reduction of approximately two times (Table 1).

**FIGURE 1:**
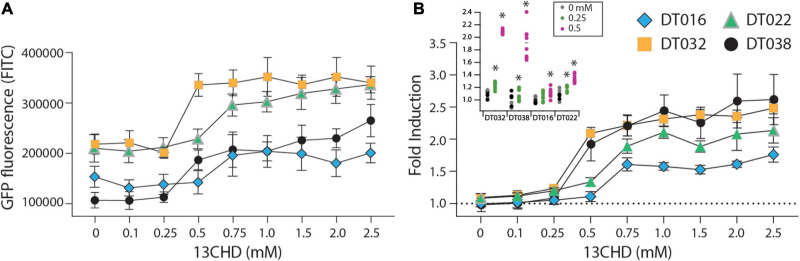
GFPmut2 fluorescence of *Escherichia coli* BW25113 Δ*rbsB* expressing DT016 (blue diamonds), DT022 (light green triangles), DT032 (ochre squares), or DT038 (black circles) in presence of different 13CHD concentrations. **(A)** Average GFPmut2 fluorescence in flow cytometry after 2 h incubation with a range of 13CHD concentrations. **(B)** As **(A)**, but as fold induction compared to uninduced conditions. Each point shows the mean of eight biological replicates (each from a measurement of 100,000 cells). Error-bars indicate calculated *SD* (not visible when inside the symbol). Dashed line represents the non-induced level. Inset in **(B)** Asterisks point to the lowest tested 13CHD concentrations giving a statistically significantly different fluorescent signal compared to the blank control (incubated in absence of any 13CHD, *p* < 0.05, two-sided *t*-test, *n* = 8 replicates).

GFPmut2 fluorescence in *E. coli* cells expressing DT016, DT022, DT032, and DT038 displayed a typical dose-dependency at different 13CHD concentrations (Figure 1). For the four mutants, the GFPmut2 fluorescence signal after 2 h induction was saturated at 0.5–0.75 mM 13CHD with 1.5–3 times fold induction (Figure 1B). Higher concentrations of 13CHD, up to 2.5 mM, did not lead to further increase of fluorescence (Figure 1A, *p* = 0.06–0.99, *t*-test equal variance, *n* = 8 replicates). The lowest concentrations of 13CHD that yielded significant induction compared to medium without inducer after 2 h incubation were 0.25 mM for DT022, DT032 and DT038, and 0.5 mM 13CHD for DT016 (Figure 1B, inset).

**FIGURE 2:**
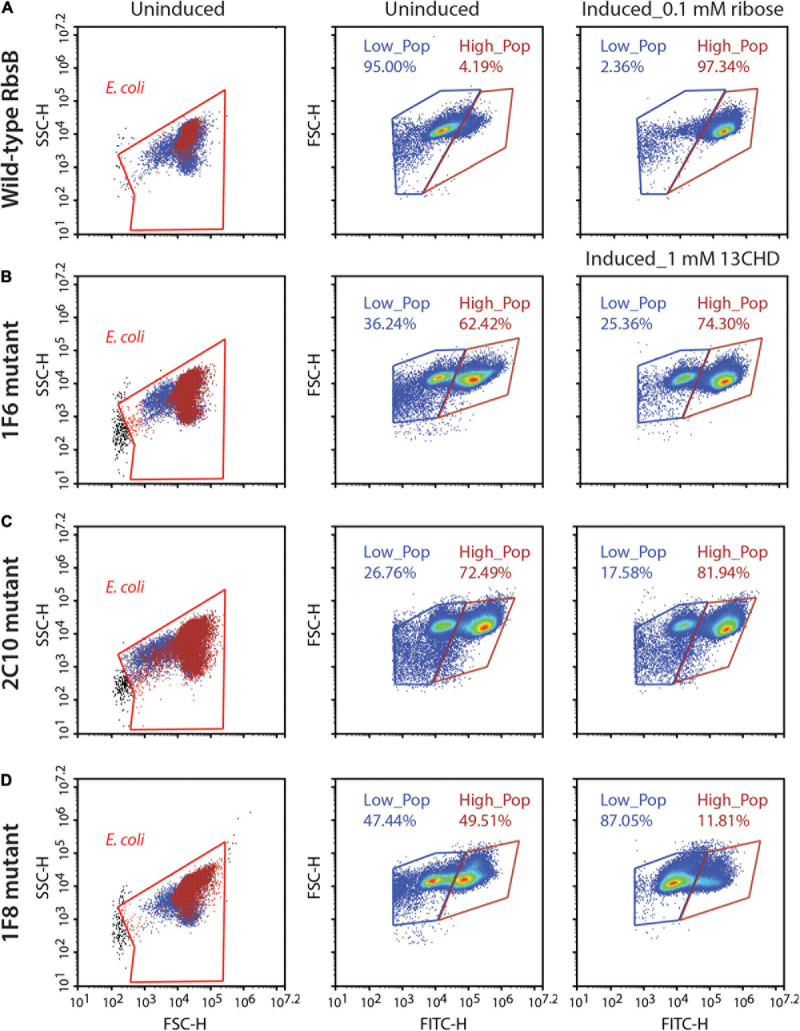
DT038-derivative mutants displaying subpopulations with two fluorescence states. Low_Pop and High_Pop represents cells with low and high fluorescence levels, respectively. Proportions of cells within both gates are indicated. **(A)** Wild-type RbsB uninduced and induced with 0.1 mM. **(B–D)** As **(A)**, but for mutant 1F6, 2C10, and 1F8 induced with 1 mM 13CHD. Each density plot shows 100,000 cells.

The four third generation (i.e., DT032, DT033, DT035, and DT038) mutants were subsequently used to create four new RMLs, which were screened as before, but this did not lead to isolation of mutant proteins with improved induction with 13CHD (i.e., more than three times GFPmut2 fluorescence increase upon induction compared to uninduced levels). We noted, however, that populations of several mutants displayed double fluorescence levels simultaneously, almost irrespectively of 13CHD presence (Figure 2). These subpopulations corresponded to completely uninduced and fully induced fluorescence levels seen from wild-type RbsB with ribose (Figure 2A, Low_Pop and High_pop). For example, mutant 1F6 displayed one subpopulation with a mean fluorescence value of 15,000 and a second of 220,000 (Figure 2B). The proportion of cells within the low and high subpopulation was approximately 36 and 62%, respectively. Upon 2 h incubation with 1 mM 13CHD the proportion of cells within either subpopulation changed to 25 and 74%, respectively (Figure 2B). Similar results were obtained with mutant 2C10, showing a reduction of 8% in the proportion of cells within the low fluorescence subpopulation upon 13CHD induction, and an increase of 9% in the high subpopulation (Figure 2C). Mutant 1F8 displayed a different behavior, with an almost equal proportion of cells distributed between the low and high subpopulations under uninduced conditions; but an increase up to 87% within the subpopulation with lowest fluorescence in presence of 13CHD (Figure 2D).

Across multiple tests and replicates, the proportions of cells within those subpopulations differed substantially, making it hard to judge whether this was consistent behavior one would expect from an inducible protein. This suggested, therefore, that these mutants had become hypersensitive and spontaneously switched between open (i.e., uninduced signal) and closed (i.e., induced) state at the level of an individual cell.

### Positions of 2nd and 3rd Generation Mutations With Improved Induction With 13CHD

**FIGURE 3:**
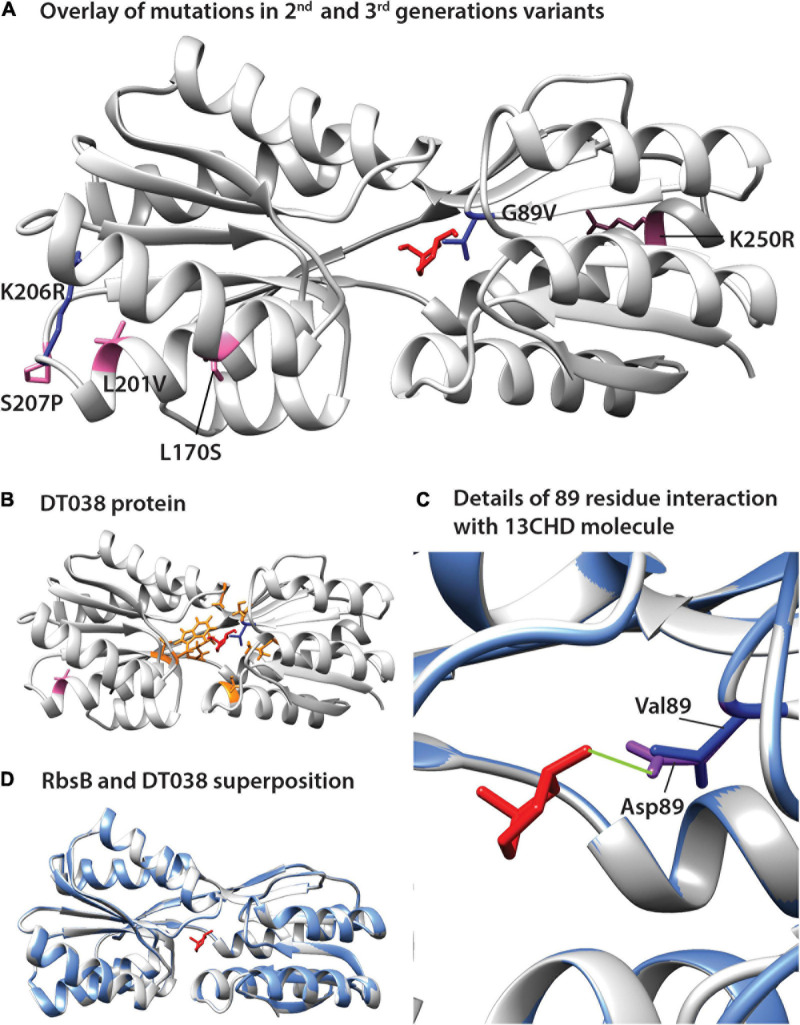
Positions of amino acid substitutions observed in various 13CHD mutants, visualized on the closed structure of wild-type RbsB (PDB ID: 2DRI). **(A)** Mutated residues in 2nd (blue) and 3rd generation mutants (pink). **(B)** Acquired mutations in DT038, seen as a superposition of 1st (orange, in DT016; the original Rosetta designed binding pocket mutations), 2nd (blue, in DT022) and 3rd generation mutations (pink). **(C)** Superposition of wild-type RbsB (PBD ID: 2DRI, blue), and the threaded DT038 structure (light gray). **(D)** Detailed view from **(C)** panel, showing the interaction between ligand and residues located at 89 position. Asp89 of RbsB and Val89 of DT038 are colored in purple and blue, respectively. Light green line indicates a H-bond. Molecular structures of 13CHD (red) are placed in the binding pocket. Signal peptide mutations are not shown on the structure as this is cleaved off.

The positions of the amino acid substitutions observed in the various new mutants (Table 1) were threaded on the closed structure of wild-type RbsB (PDB ID: 2DRI). Six out the seven isolated mutants displayed a single amino acid substitution. Exception was DT033 that displayed three amino acid changes. Mutant DT020 displayed a conservative substitution within the signal peptide (V10I) (Table 1). DT021 and DT022 displayed conservative amino acid substitutions K206R and G89V, respectively (Figure 3A and Table 1). The V89 residue of DT022 is located within the binding pocket, 1.9 Å from the 13CHD molecule (Figure 3A). This suggests that the G89V substitution is directly responsible for the 50% increase of GFPmut2 fluorescence upon 13CHD induction, when compared to its parent DT016 (Tavares et al., 2019). Previous studies demonstrated the importance of residue 89 for ligand binding (Vercillo et al., 2007; Reimer et al., 2017; Tavares et al., 2019), suggesting that V89 improves the capacity to bind 13CHD in comparison with G89. Finally, mutation K206R found in DT021 is located in a peripheral turn of the structure (Figure 3A).

Two out of the four 3rd generation mutants displayed non-conservative mutations. Notably, DT032 had a leucine at position 170 instead of serine, whereas DT033 displayed a S207P substitution. DT033 showed two conservative mutations (L201P and K250R; Figure 3A and Table 1). Mutant DT035 again displayed a mutation in the signal peptide (K5N). This and the V10I substitution of DT020 in the signal peptide may have improved the translocation and/or stability of the mutant protein. Of the other five substitutions observed in this 3rd round of evolution, three (L170S, L201V, and K250R) were located outside the binding pocket in three different α-helices of the protein (Figure 3A). The two others (K206R and S207P) were localized in a peripheral turn of the protein structure (Figure 3A). All of them led to an increase of the fold–induction with 13CHD (Table 1). However, their peripheral position suggested they play a role in protein stability and not directly in ligand binding. Interestingly, five out the seven substitutions found in 2nd and 3rd generation mutants localized in the same region of the protein (Figure 3A), suggesting that changes in that area improve protein function (e.g., stability or intramolecular hinge movement). Interestingly, leucine at position 201 was substituted twice independently by two different amino acids (i.e., proline and valine), underscoring its critical role. None of the positions recovered in these DT variants for 13CHD binding had been previously described as critical for the various roles of RbsB in a near-complete Ala-substitution scanning (Reimer et al., 2017).

To further infer potential structural changes of observed mutants in comparison to wild-type RbsB, we used Swiss-Model (Guex et al., 2009; Bertoni et al., 2017; Bienert et al., 2017; Waterhouse et al., 2018; Studer et al., 2020), Phyre2 (Kelley et al., 2015) and Missense3D (Ittisoponpisan et al., 2019). Swiss-Model, Phyre2 did not predict any structural differences of the new mutations compared to the closed structure of wild-type RbsB (Figures 3B,C, shown for DT038). Analysis of each of the eight amino acid substitutions in DT016 by Missense3D indicated expansion of the binding cavity by F16S and R90S, and H-bond breakage by D89V and T135A. This was expected, since these were designed and engineered ligand binding pocket mutations to accommodate 13CHD. However, none of other four mutations in DT016 were predicted by Missense3D to cause any (individual) structural difference compared to RbsB. Interestingly, the G89V mutation in DT022 compared to DT016 (or D89V compared to wild-type) was predicted to cause further expansion of the ligand binding pocket and to H-bond breakage (Figure 3D). This is probably the consequence of replacing a buried amino acid (Gly) by an exposed one (Val). Other individual amino acid substitutions, found in other isolated mutants, were not predicted to cause any structural difference compared to wild-type RbsB, but we acknowledge that Missense3D only tests single substitutions at a time.

### DNA Shuffling and Site Saturation Mutagenesis

Rescreening of the 2nd round RML002 and RML016 libraries, led not only to the isolation of the second generation mutants (i.e., DT020, DT021, and DT022) but to eight more variants as well (Table 2). Individual retesting of those eight variants showed no change in the mean fold-induction of GFPmut2 in presence of 1 mM 13CHD compared to DT016 itself (Table 2, *p* = 0.245–0.89, *n* = 6 replicates). On the other hand, five mutants (named here: 2H2, 7B2, 7B9, 7C5, and 7G4) displayed a lower background fluorescence in uninduced conditions, when compared to DT016 (Table 2, *p* = 0.01–0.00001, *n* = 6 replicates). The highest background reduction was two-fold, observed in 7G4 mutant (Table 2). The lower fluorescence background suggests a better equilibrium between open and closed conformation. The DNA of the eight mutants was then shuffled in the hope to create synergetic effects, but no mutant with improved induction by 13CHD was isolated from this screening.

**TABLE 2:** List of isolated mutants from RML002 and RML016 2nd round used for DNA shuffling.

**RbsB protein**	**GFPmut2 uninduced fluorescence^a^**	**Fold induction^b^ 1 mM 1,3-cyclohexanediol**	**New mutation(s)^c^**
Wild-type	**25,226 ± 4,066^d^**	**0.91 ± 0.05**	–
DT016	160,622 ± 33,495	1.87 ± 0.26	^e^
2H12	**112,793 ± 16,657**	2.01 ± 0.13	Q80R
3B11	157,805 ± 24,521	1.88 ± 0.13	V17E T58A
5H2	128,273 ± 13,989	1.97 ± 0.11	K29R A214T
6H9	132,217 ± 12,670	1.92 ± 0.1	I132T
7B2	**95,166 ± 8,064**	1.97 ± 0.11	T93M
7B9	**91,342 ± 1,4436**	1.75 ± 0.06	T10A
7C5	**85,631 ± 15,553**	1.89 ± 0.14	N175S K228Q T232D
7G4	**78,653 ± 8,090**	1.81 ± 0.16	N73S

*^*a*^Mean ± one *SD* GFPmut2 fluorescence values of uninduced cultures (*n* = 6–8).*

*^*b*^Mean fold induction ± one *SD*, as the ratio of the mean GFPmut2 fluorescence of induced cultures with 1 mM 13CHD and the mean fluorescence of uninduced cultures.*

*^*c*^Newly observed mutations in comparison to DT016.*

*^*d*^Values in bold are statistically significantly different from those of DT016 (*p* < 0.05, *t*-test, equal variance).*

*^*e*^DT016 carries eight mutations compared to wild-type RbsB: F16S, N64V, D89V, R90S, T134A, F164W, F214A, and Q235M.*

**FIGURE 4:**
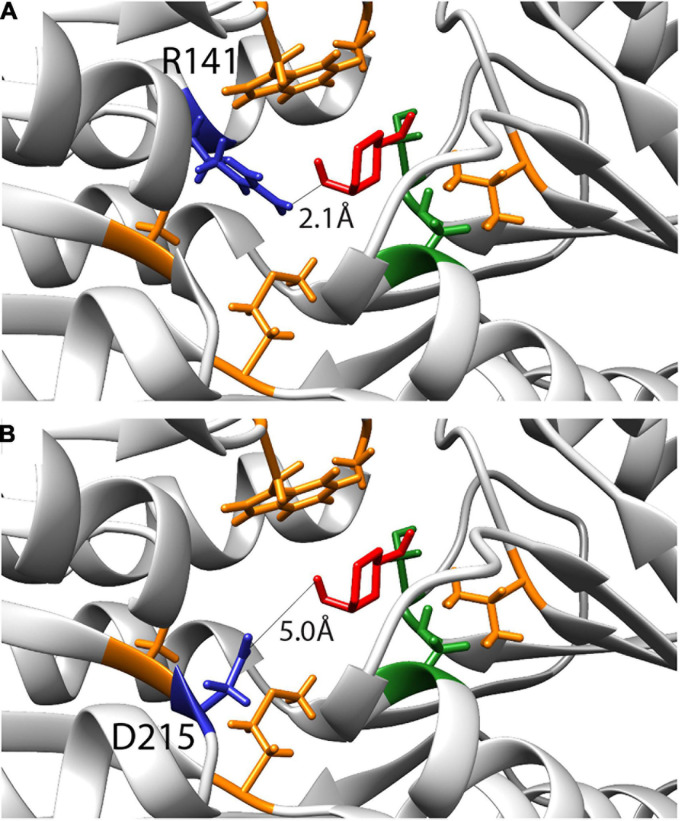
Inferred binding pocket of DT016 in presence of 13CHD (in red) and with the substituted residues for ligand binding color-coded based on characteristics (non-polar- orange; polar- green). **(A)** Arginine at position 141, selected for site saturation mutagenesis, is highlighted in dark blue. Distance between 13CHD molecule and R141 is indicated. **(B)** As for **(A)** but for Aspartic acid at position 215.

Computational simulations had previously suggested nine amino acids as being critical for changing the specificity of RbsB protein to 13CHD (Tavares et al., 2019). Two residues were later found by ala-substitution scanning to be important for ribose binding (R141 and D215) (Reimer et al., 2017). We therefore tested whether site-saturation mutagenesis of these residues could further improve DT002 and DT016 variants for 13CHD induction (Figure 4). Replacement of R141 and D215 by each of the other 20 possible amino acids was confirmed by sequencing and 250 colonies of each site saturation library were tested individually by flow cytometry for gain of 13CHD induction. None of the tested mutants from the DT002_R141X, DT002_D215X, or DT016_R141X and DT016_D215X libraries showed improved 13CHD induction, compared to parental strains, whereas several were worse. *Inter alia*, this showed that R141 in mutant DT016 can be replaced by a serine without impairing inducibility by 13CHD.

### Mutagenesis of Neighboring Residues in DT016

**FIGURE 5:**
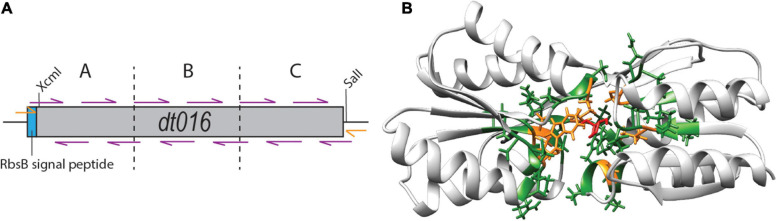
Flanking residue mutagenesis. **(A)** Scheme of the design strategy to reconstitute *dt016* with 12 overlapping primers (purple), positioned at the sites of the previous ligand binding pocket mutations, and each covering the two neighboring codons on each side. The assembly was carried out in three steps (fragments separated by dotted lines) and followed by a final extension reaction with external primers (orange). Gene not drawn to scale. **(B)** Inferred structure of DT016 based on RbsB structure (PDB ID: 2DRI). Molecular structure with 13CHD (red) bound in its pocket. The eight designed substitutions in DT016 are highlighted in orange and flanking residues mutated with this strategy are highlighted in green.

Finally, we tested whether substitutions in the direct neighborhood of the previously engineered ligand binding pocket mutations would affect induction by 13CHD, through synergistic or compensatory effects on the overall protein function or behavior. For this we focused again on DT016, the most promising mutant with newly obtained specificity to 13CHD (Tavares et al., 2019). Next, we designed a strategy to mutate the two amino acids flanking (i.e., those before and after) each of the eight ligand binding pocket mutations of DT016 (Supplementary Figure 2). We reconstituted the *dt016* open reading frame in 12 overlapping PCR fragments (Figure 5A). PCR primers covered the regions of the eight introduced mutations in DT016 (Figure 5B), with flanking amino acids of those being replaced by all other 20 possible amino acids (Table 3). The disadvantage of this strategy was that stop codons could not be avoided in primer design. A library with an estimated size of 1 × 10^6^ clones (RML-DT016AA) was screened as before by agarose encapsulation and flow cytometry. As expected, a large fraction of clones carried truncated proteins (75% from 25 randomly picked colonies from the library on plates). None of the clones displayed higher fold induction than DT016 itself. We concluded that this strategy was not worth further pursuing.

**TABLE 3:** Strains used in this study.

**Strain**	***E*. *coli* host**	**Plasmids**	**Relevant characteristics**	**References or sources**
3044	DH5αλPir		Host for plasmid propagation	Platt et al. (2000)
3671	DH5α	pSTVPAA_mcs	pSTVPAA to clone rbsB and its derivatives	Reimer et al. (2014)
4172	BW25113 ΔrbsB	pSYK1	Host strain containing the Ptac- trzI, PompC- gfpmut2 bioreporter system	Reimer et al. (2014)
4175	BW25113 ΔrbsB	pSTVPAA_rbsB, pSYK1	RbsB expression with signal peptide for periplasmic translocation	Reimer et al. (2014)
5913	BW25113 ΔrbsB	pSTVPAA_DT001, pSYK1	As 4175, but for DT001 mutant protein of RbsB	Tavares et al. (2019)
5903	BW25113 Δ*rbsB*	pSTVPAA_DT002, pSYK1	As 4175, but for DT002 mutant protein of RbsB	Tavares et al. (2019)
5904	BW25113 Δ*rbsB*	pSTVPAA_DT011, pSYK1	As 4175, but for DT011 mutant protein of RbsB	Tavares et al. (2019)
5905	BW25113 Δ*rbsB*	pSTVPAA_DT013, pSYK1	As 4175, but for DT013 mutant protein of RbsB	Tavares et al. (2019)
5906	BW25113 Δ*rbsB*	pSTVPAA_DT015, pSYK1	As 4175, but for DT015 mutant protein of RbsB	Tavares et al. (2019)
5907	BW25113 Δ*rbsB*	pSTVPAA_DT016, pSYK1	As 4175, but for DT016 mutant protein of RbsB	Tavares et al. (2019)
7241	BW25113 Δ*rbsB*	pSTVPAA_DT020, pSYK1	As 4175, but for DT020 mutant protein of RbsB	This work
7242	BW25113 Δ*rbsB*	pSTVPAA_DT021, pSYK1	As 4175, but for DT021 mutant protein of RbsB	This work
7243	BW25113 Δ*rbsB*	pSTVPAA_DT022, pSYK1	As 4175, but for DT022 mutant protein of RbsB	This work
7244	BW25113 Δ*rbsB*	pSTVPAA_DT032, pSYK1	As 4175, but for DT032 mutant protein of RbsB	This work
7245	BW25113 Δ*rbsB*	pSTVPAA_DT033, pSYK1	As 4175, but for DT033 mutant protein of RbsB	This work
7246	BW25113 Δ*rbsB*	pSTVPAA_DT035, pSYK1	As 4175, but for DT035 mutant protein of RbsB	This work
7247	BW25113 Δ*rbsB*	pSTVPAA_DT038, pSYK1	As 4175, but for DT038 mutant protein of RbsB	This work
2H12	BW25113 Δ*rbsB*	pSTVPAA_2H12, pSYK1	As 4175, but for 2H12 mutant protein of RbsB	This work
3B11	BW25113 Δ*rbsB*	pSTVPAA_3B11, pSYK1	As 4175, but for 3B11mutant protein of RbsB	This work
5H2	BW25113 Δ*rbsB*	pSTVPAA_5H2, pSYK1	As 4175, but for 5H2 mutant protein of RbsB	This work
6H9	BW25113 Δ*rbsB*	pSTVPAA_6H9, pSYK1	As 4175, but for 6H9 mutant protein of RbsB	This work
7B2	BW25113 Δ*rbsB*	pSTVPAA_7B2, pSYK1	As 4175, but for 7B2 mutant protein of RbsB	This work
7B9	BW25113 Δ*rbsB*	pSTVPAA_7B9, pSYK1	As 4175, but for 7B9 mutant protein of RbsB	This work
7C5	BW25113 Δ*rbsB*	pSTVPAA_7C5, pSYK1	As 4175, but for 7C5 mutant protein of RbsB	This work
7G4	BW25113 Δ*rbsB*	pSTVPAA_7G4, pSYK1	As 4175, but for 7G4 mutant protein of RbsB	This work

## Discussion

Periplasmic binding proteins have been deployed as a starting point to design new receptor proteins (Dwyer and Hellinga, 2004). Despite the vast knowledge on PBPs structures and their natural ligands (Berntsson et al., 2010), the successful design of new ligand binding domains has been very limited so far (Schreier et al., 2009; Yang and Lai, 2017). Introducing amino acid substitutions in a protein is challenging, since they can easily lead to an abnormal function or behavior of the mutated protein (Reimer et al., 2017). In a previous study nine residues were identified and substituted in the binding pocket of RbsB, with the goal to change the binding specificity from the natural ligand ribose to the non-natural compound 13CHD (Tavares et al., 2019). Despite the modest increase of induction (up to 1.5-fold with 13CHD), six mutant proteins without ribose recognition but with 13CHD binding were isolated. Relatively poor induction had been attributed to mutant protein instability, poor translocation, and/or misfolding (Tavares et al., 2019).

The goal here was to produce and select compensatory mutations by random or semi-random approaches, which might either have a stabilizing effect or further improve 13CHD ligand binding, or both. We focused on the previously isolated mutants, which we used as scaffolds for mutagenesis. Several rounds of random mutagenesis and increasing selectivity of sorting of bead-grown microcolonies induced with 13CHD, led to recovery of a few mutants with consistently higher induction of GFPmut2 fluorescence than their parental strains (up to 3.2-fold at 1 mM 13CHD). As these mutants carried mostly substitutions outside the direct ligand binding pocket, we assume that they are compensatory mutations that improve functions other than ligand binding itself, for example, L170S in DT032 or L201V in DT038.

In order to maximize our chances to isolate an improved variant for 13CHD detection, we used different mutagenesis approaches to create genetic variability. Our semi-random approaches did not produce the expected results, since no improved variant was isolated from the created libraries. Site saturation mutagenesis of R141 and D215 residues on DT002 and DT016 resulted in decrease of the capacity for induction by 13CHD, except for a R141S substitution in DT016 that did not affect inducibility. This indicated that we could not improve the 13CHD induction by replacing R141 and D215 residues; in contrast, it showed that their presence is essential for 13CHD binding and signaling. The importance of both R141 and D215 residues in RbsB for ribose induction and signaling (Reimer et al., 2017) and for ligand binding (D215) had been previously demonstrated (Vercillo et al., 2007). Also DNA shuffling did not lead to isolation of mutants with potential synergistic improvements, although background reduction in absence of inducer was observed (Table 2). Random mutation of the 32 residues flanking the nine substitutions engineered for 13CHD ligand binding did not yield improved variants either, possibly because of the high percentage of variants with a truncated protein. Some variants displayed a comparable induction level to their parent DT016 (around 1.5 times). However, given the high number of substitutions in these variants (up to 32 amino acid substitutions), an interpretation of their effect was impossible. These results indicated that changing several parts of the RbsB-mutant proteins at the same time may not be the best way to find variants with improved function. Introducing multiple mutations increases the probability to find proteins with improved capacities, but at the same time increases the chances to introduce mutations that may impair the protein function. This creates an important trade-off, and has to be considered each time when designing and implementing a mutagenesis strategy.

In contrast, random mutagenesis across the complete gene variants led to the isolation of seven mutants with significantly improved 13CHD inducibility, two of which with 4–4.5-fold improvement of induction. This was accomplished by screening of relatively large libraries on microcolonies grown in beads, under different screening thresholds and several rounds of repetition. We acknowledge that FACS thresholding in such screening is a difficult point, because distinguishing between fluorescence outliers of false-positive clones and true positive inducible ones can be subjective. In less restrictive sortings, all beads above the 98th fluorescence percentile of the 13CHD induced library were collected, re-used as template for a new library, from which we recovered the top 0.1% fluorescence beads. This strategy led to isolation of three variants with improved induction with 13CHD. In the more restrictive sorting, only beads with a fluorescence higher than any bead under uninduced conditions were recovered. This resulted in isolation of four mutants with up to 3.2-fold induction by 13CHD. Since both strategies allowed us to isolate mutants with improved 13CHD detection, we conclude that the restrictive sorting is a better strategy, partly because of the time investment and downstream screening of individual clones. A disadvantage of the restrictive strategy is that mutants are missed that have low fluorescence background under uninduced conditions and intermediate fluorescence upon induction (i.e., a fluorescence signal less than the maximum observed in the uninduced library). Alternatively, one could try to “bin” mutants in different fluorescence categories in the hope of finding some with lower fluorescence backgrounds and still some induction. The difficulty is that *a priori* the evolutionary path of a variant highly inducible by 13CHD is not known and may pass through intermediates with high uninduced levels to regain background, or through those with low uninduced levels and gain specificity (Romero and Arnold, 2009; Tracewell and Arnold, 2009; Zheng et al., 2020). Multiple rounds of mutagenesis thus allowed to improve 13CHD detection in a step-by-step manner. This suggests that further rounds of random mutagenesis could eventually lead to the isolation of a variant with similar binding capacity to 13CHD as wild-type RbsB toward ribose (13 fold) (Tavares et al., 2019), although we could not achieve that here. Some studies show that multiple rounds of evolution are needed to improve a specific protein ability without impairing the protein (Brustad and Arnold, 2011).

What can we conclude from the obtained DT variants in terms of amino acid substitution effects? Two mutants (DT020 and DT035) displayed an amino acid substitution in the signal peptide (V10I and K5N). The improved 13CHD induction might have been due to higher periplasmic protein levels, being the result of a positive effect on peptide recognition by SecB chaperone, responsible for presenting RbsB to the translocation channel, and or improved stability. Only one variant (DT022) carried a substitution (G89V) in the binding pocket (Figures 3A,D). This residue is less than 2 Å away from the inferred position of 13CHD and previous studies demonstrated the importance of residue 89 for ligand binding (Vercillo et al., 2007; Reimer et al., 2017; Tavares et al., 2019). An exposed valine residue at this position thus might improve 13CHD binding, yielding a 50% higher fold induction when compared with parent DT016 (Table 1). All other mutations were found outside the binding cavity, and we assume that they must have improved other aspects of protein functionality than ligand binding itself, although we did not test this specifically by biochemical methods on purified protein. This could affect, for example, protein stability or improved hinge flexibility, or binding to the chemoreceptor Trz1. Five out the seven mutations were located in the same peripheral region of the protein (Figure 3A), but none concerned positions previously implicated in RbsB functioning by Ala-substitution scanning (Reimer et al., 2017). Leucine at position 201 was replaced by two other non-polar residues in two different isolated mutants and neighboring residues K206 and S207 were replaced by arginine and proline, respectively. The concentration of observed mutations in this region suggests that previous introduced mutations may have disturbed this region of the protein and compensatory mutations were needed. This specific region of the protein, therefore, could be a promising target for future rounds of mutagenesis, aiming to find variants with better overall function. Importantly, the new variants were not only more highly induced by 13CHD, but also displayed reduced fluorescence background, especially DT032, DT035, and DT038. This is further evidence that these mutations are compensatory and improve the overall functionality of the proteins in the bioreporter signaling cascade.

Creation of new ligand-binding cavities in PBPs had been heralded more than a decade ago as one of the key areas of advance for computational protein design (Looger et al., 2003), but more recent *de novo* design of protein (and peptide) structure design have focused more on small-molecule-binding proteins (Polizzi and DeGrado, 2020), switchable/allosteric capacity (Langan et al., 2019), protein folding (Rocklin et al., 2017) and epitope-scaffolds design (Sesterhenn et al., 2020). Much of the initial claimed successes of PBP ligand pocket engineering has not held up under the scrutiny of independent repetitions (Schreier et al., 2009; Reimer et al., 2014). More recent advances have been reported that have shown grafting of existing ligand pockets in PBPs, and a single study of a *de novo* design achieving marginal 13CHD binding (Scheib et al., 2014; Banda-Vazquez et al., 2018; Tavares et al., 2019). It might thus well be that, in contrast to the original assumption of a wide protein family with known crystal structures of open and closed configurations, PBPs are actually particularly difficult to engineer. The reasons may be that PBPs need an inherent intramolecular protein movement between open and closed configuration and have manifold functional constraints, such as ligand binding, binding to the receptor, or translocation. Current ligand pocket predictions do not take the other constraints into consideration, which make complete rational computational design challenging.

For example, in the RbsB-based bioreporter configuration wild-type and mutant-RbsB proteins have to be expressed and translocated to the periplasmic space. Once in the periplasmic they recognize and bind their ligand, leading to a conformational change of the protein (Boas and Harbury, 2008; Stank et al., 2016). Subsequently, the closed form of the protein binds the hybrid Trz1 receptor, starting a phosphorylation cascade that in the end leads to induction of GFPmut2 expression (Reimer et al., 2014). It is important to understand that if an introduced mutation affects any aspect of these steps the final outcome (i.e., GFPmut2 signal) is affected. The transition between open and closed conformation is extremely important for PBPs with bilobal structure such as RbsB. It is assumed that PBPs in absence of ligand can be found in a dynamic equilibrium of open and closed state (Ravindranathan et al., 2005; Schreier et al., 2009), which is important for their function. Similar characteristics are observed in other PBPs, for example in the closely related galactose-binding protein of *E. coli* (Unione et al., 2016). In presence of the proper ligand, the closed form is stabilized (Schreier et al., 2009) and, like in case of RbsB can present the ligand molecule (ribose) to either the chemoreceptor (i.e., Trg and Trz1) or to ribose transport channels (Binnie et al., 1992; Riley, 1993; Stewart and Hermodson, 2003). We observed that introduced mutations can block RbsB variants in either of the two states, and consequently, disable its function to bind the ligand and trigger the bioreporter system, or trigger the receptor signaling cascade without binding the ligand. We also observed RbsB-DT variants that in the *E. coli* bioreporter strain caused “stable” double populations with different GFPmut2 fluorescence intensities both in absence and in presence of inducer. This is in contrast to wild-type RbsB behavior, which (despite reported open-closed form dynamics in absence of ligand) in absence of ribose results in coherent low reporter output and in the presence of ribose in coherent high fluorescence. This suggests that the time-scale of the dynamics may be affected by the introduced mutations, blocking the DT variants in either open or closed form long enough to trigger (or not) the bioreporter signaling cascade leading to GFP expression. This is supported by the fact that these subpopulations corresponded to completely uninduced and fully induced fluorescence levels seen from wild-type RbsB with ribose (Figure 2). A small percentage of the low fluorescence population shifts to high fluorescence upon induction, indicating that ligand-binding is still affecting the transition states, but is insufficiently discriminating between the two (Figures 2B,C). The implications of individual and combinations of secondary mutations for specific aspects of RbsB-DT variant functionality, e.g., translocation, stability, intermolecular movement, folding, ligand or receptor binding, however, can only be derived from more precise biochemical techniques with purified protein.

In conclusion, the results obtained in this study showed that it is possible to improve the signaling performance of previously designed RbsB mutants with *de novo* ligand binding pockets using random mutagenesis. The two most promising mutants DT032 and DT038 displayed a 4–4.5-fold improvement in induction to 13CHD, mostly as a result of background reduction in absence of inducer (Table 1). The variants react in a dose-dependent manner, with a lower detection limit around 0.25 mM 13CHD. This study demonstrates the principle that new PBP ligand-binding domains can be engineered using the RbsB signal transduction bioreporter platform and that more work is needed to achieve a ligand detection limit that approaches the wild-type RbsB sensor for ribose, which demonstrates a 13-fold fluorescence induction and 50 nM detection limit (Reimer et al., 2014; Tavares et al., 2019).

## Materials and Methods

### Bacterial Strains and Culture Conditions

Expression of the RbsB-Trz1-*ompCp-gfpmut2* signaling chain (or the RbsB variants) was tested in *E. coli* BW25113 Δ*rbsB* as host. In this case, cells were cultured in minimal medium (MM) supplemented with 20 mM fumarate. For selection of mutants by FACS, cells were first grown within alginate beads in low phosphate minimal medium (MM LP) supplemented with 20 mM fumarate and appropriate antibiotics to produce microcolonies, as described previously (Tavares et al., 2019). The cells-in-beads were then induced with 0.1 mM ribose or 1 mM 1,3-cyclohexanediol (13CHD) for 2 h, as described previously (Tavares et al., 2019). *Escherichia coli* DH5α cells were used for cloning and plasmid propagation. Random libraries were transformed into ElectroMAX DH10B T1 Phage-Resistant competent cells (Thermofisher).

All strains used in this study are listed in Table 3.

### Random Mutagenesis Libraries and Plasmid Construction

Mutations in the *rbsB* gene or its *dt* variants were generated by error-prone PCR (ep-PCR). Gene variants were amplified by primers flanking the coding sequence in the plasmids pSTVPAA-DTxxx and located up- and downstream of the *Sal*I and *Nde*I sites (Supplementary Figure 1). Error-prone-PCR reactions were carried out with 4 ng of DNA template in presence of varying MnCl_2_ concentrations (0.025–0.06 mM). Six reactions were prepared simultaneously to average stochastic biases. After an initial denaturation step of 10 min at 94°C, the following steps were repeated for 25 cycles: 1 min at 94°C, 1 min at 70°C, and 1.5 min at 72°C, followed by an extension of 10 min at 72°C. Amplicons were then visualized by agarose electrophoresis, and products of around 1 kb were excised, pooled and purified. Purified PCR products and pSTVPAA plasmids were digested with *Sal*I and *Nde*I at 37°C and 300 rpm for 45 min. Plasmid self-ligation was prevented by treating the digested plasmid with Shrimp Alkaline Phosphatase (rSAP, New Englang Biolabds). The digestion products were visualized by agarose electrophoresis, and the correctly sized digested bands were excised from the gel and purified.

Plasmid and amplicon fragments were ligated with T4 DNA ligase using a ratio of 1:2 vector to insert. The ligation mixture was incubated overnight at room temperature, and aliquots of 100 ng DNA were electro-transformed into ElectroMAX DH10B cells. Cells were recovered after the electroporation by addition of 1 mL of SOC medium, pooled from five separate reactions and incubated for 1 h 30 at 37°C, 225 rpm. Small proportions of these mixtures where plated on LB agar (Cm, chloramphenicol at 30 μg ml^–1^) plates to estimate the number of colony forming units in the libraries. The remainder was cultured *en masse* in 200 ml LB medium (with Cm), which was used to isolate and purify a plasmid-library pool. Aliquots of 1.6 mL of the grown culture were stored in 15% *(v/v)* glycerol at –80°C. Five aliquots of each 100 ng of purified pSTVPAA-mutant plasmid pool were then transformed into the bioreporter strain *E. coli* BW25113 Δ*rbsB*, for testing of ribose- and 13CHD-dependent expression of GFPmut2. Library aliquots were again stored at –80°C. Error-prone-PCR in presence of different MnCl_2_ concentrations (0.025–0.06 mM) resulted in between 1 and 3 mutations per 1,000 bp.

### DNA Shuffling and Site Saturation Mutagenesis

As an alternative to error-prone-PCR we used DNA shuffling to create new *rbsB* variants. For this we used eight *rbsB* variants as template (Table 2), which were amplified by PCR using primers outside the coding regions and beyond the *Xcm*I and *Sal*I sites (Supplementary Figure 1 and Table 4). PCR-amplified templates (200 ng each) were mixed and digested with 0.5 U of DNase I for 3 min at 15°C, after which the reaction was inactivated at 80°C for 10 min. Aliquots of 200 ng of fragmented DNA were then reassembled by PCR in progressive hybridization in presence of 2.5 U of GoTaq polymerase (Thermofisher) and 200 μM of each dNTP in the following temperature cycles. After an initial denaturation period of 2 min at 94°C, the following steps were repeated for 35 cycles: 40s at 94°C, 90s from 65 to 41°C in intervals of 3°C and 90s at 68°C, followed by a final 30 min period at 68°C. The PCR reassembly products were next amplified with primers located inside the previous ones (Supplementary Figure 1 and Table 4), visualized by agarose electrophoresis, after which 1-kb DNA bands were isolated and purified. These products were digested with *Xcm*I and *Sal*I and ligated to pSTVPAA digested with the same enzymes. Ligation mixture aliquots of 100 ng each were then transformed into the bioreporter strain *E. coli* BW25113 Δ*rbsB*.

**TABLE 4:** List of primers used to introduce new mutations in RbsB-derivate proteins.

**Mutagenesis Technique**	**Target**	**Primers**	**DNA sequence (5′–3′)**
DNA shuffling	*rbsB* (outer primers)	190101 F	CAGCTGGCGAAAGGGGGATGTG
		130401 R	CTGAGCACATCAGCAGGAC
	*rbsB* (inner primers)	160401 F	CACGACGTTGTAAAACGACGGCC
		190102 R	CTGGCTACCCTGGTTTCCGCTG
Site saturation mutagenesis	pSTVPAA-DT002R141X	180901 F	GAAGCCTTCGCCNNNTTCACGGGCTGCGGACGCACC^*a*^
		180702 R	GCAGCCCGTGAANNNGGCGAAGGCTTCCAGCAGGCC
	pSTVPAA-DT002D215x	180705 F	ATCCGGTGTACCNNNAGCTCCGACGACCATCACATC
		180706 R	GTCGTCGGATCGNNNGGTACACCGGATGGCGAAAAA
	pSTVPAA-DT016R141X	180901 F	GAAGCCTTCGCCNNNTTCACGGGCTGCGGACGCACC
		180702 R	GCAGCCCGTGAANNNGGCGAAGGCTTCCAGCAGGCC
	pSTVPAA-DT016D215x	180901 F	ATCCGGTGTACCNNNCGCTCCGACGACCATCACATC
		180708 R	GTCGTCGGAGCGNNNGGTACACCGGATGGCGAAAAA

*^*a*^NNN indicates mutated positions for production of the libraries.*

Positions R141 and D215 in the DT002 and DT016 were changed by site-saturation mutagenesis. For this, the entire plasmid(s) pSTVPAA-DT002 or −016 (5 ng) was amplified by Q5 High-Fidelity DNA polymerase using overlapping but reverse complementary primers with ambiguous bases at the desired positions (Figure 4 and Table 4). PCR products were digested with *Dpn*I to remove template DNA and after enzyme inactivation were directly transformed into the bioreporter strain *E. coli* BW25113 Δ*rbsB*. Variant genes were confirmed by sequencing.

### Mutagenesis of Neighboring Residues in DT016

In order to reconstitute the *rbsB* gene 12 overlapping primers were designed (Table 5). Assembly of the 12 designed primers was carried out in two steps. In the first step, primers were divided in three independent annealing groups (Figure 5A). PCR for every group was performed with 200 μM of dNTPs, 50 nM of each primer and 0.02 U/μL of Q5 High-Fidelity DNA polymerase. After an initial denaturation period of 30 s at 98°C, the following steps were repeated for 10 cycles: 10 s at 98°C, 15 s at 45°C, and 20 s at 72°C, followed by a final 2 min period at 72°C. In the second step, 2 μL of the three independent reactions were mixed with 200 μM of dNTPs and 0.02 U/μL of Q5 High-Fidelity DNA polymerase (same PCR conditions as in step1). PCR products were then finally extended with external primers, 1 μL of the 2nd assembly reaction was amplified by PCR and reactions were performed in triplicate to remove any bias. After an initial denaturation period of 30 s at 98°C, the following steps were repeated for 35 cycles: 10 s at 98°C, 30 s at 68°C, and 30 s at 72°C, followed by a final 5 min period at 72°C. Amplified products were visualized by agarose electrophoresis and 1 kb DNA bands were isolated and purified. Final products were digested with *Xcm*I and *Sal*I and ligated to pSTVPAA digested with same enzymes. After overnight ligation with T4 DNA ligase, D5Hα cells were transformed with 100 ng of DNA for plasmid replication. After rescue a small percentage of cells were plated and were sent for sequencing to estimate library size and variability.

**TABLE 5:** List of primers used to introduce new mutations close to pre-existing mutations on DT016 protein.

**Primers**	**Target, assembly reaction**	**DNA sequence (5′–3′)**
190201 For	*dt016* gene, 1st assembly - reaction A	ATGGCAAAAGACACCATCGCGCTGGTGGTCTCCACGCTTAACAACNNNNNNAGCNNNNNNCTGAAA GATGGCGCGCAG^*a*^
190202 Rev	*dt016* gene, 1st assembly - reaction A	TCGCCGGGTTGTTCTGGGAGTCCAGCACCACCAGGTTATAGCCAAGTTTATCCGCCTCTTTCTGCGC GCCATCTTTCAGNNNNNNGCTNNNNNNGTTGTTAAGCGTGGAG
190203 For	*dt016* gene, 1st assembly - reaction A	AGAACAACCCGGCGAAAGAGCTGGCGAACGTGCAGGACTTAACCGTTCGCGGCACAAAAATTCTGN NNNNNGTGNNNNNNGACTCC
190204 Rev	*dt016* gene, 1st assembly - reaction A	ACCTTTCGTNNNNNNGCTGCCNNNNNNGATAACCGGGATGTTCGCCTGGTTAGCCATCTTCACAGCA TTACCCACTGCGTCGGAGTCNNNNNNCACNNNNNNCAGAATTTTTGT
190205 For	*dt016* gene, 1st assembly - reaction B	TCCCGGTTATCNNNNNNGGCAGCNNNNNNACGAAAGGTGAAGTGGTGAGCCACATTGCTTCTGATAA CGTACTGGGCGGCAAAATCGCTGGTGATTACATCGCG
190206 Rev	*dt016* gene, 1st assembly - reaction B	ACGTTCACGGGCNNNNNNCGCNNNNNNAATGCCTTGCAGCTCGATAACTTTGGCACCTTCACCCGC TTTCTTCGCGATGTAATCACCA
190207 For	*dt016* gene, 1st assembly - reaction B	AGGCATTNNNNNNGCGNNNNNNGCCCGTGAACGTGGCGAAGGCTTCCAGCAGGCCGTTGCTGCTC ACAAGTTTAATGTTCTTGCCAGCCAGCCANNNNNNTGGNNNNNNATTAAAGGT
190208 Rev	*dt016* gene, 1st assembly - reaction B	CCCAGCGCCATNNNNNNGTTNNNNNNGAATACAGCCTGAACATCCGGATGAGCGGTCAACAGGTTC TGCATTACGTTCAAACCTTTAATNNNNNNCCANNNNNNTGGCTGGCTGGC
190209 For	*dt016* gene, 1st assembly - reaction C	GGCTGTATTCNNNNNNAACNNNNNNATGGCGCTGGGCGCGCTGCGCGCACTGCAAACTGCCGGTA AATCGGATGTGATGGTCNNNNNNGCGNNNNNNACACCGGATGGCGA
190210 Rev	*dt016* gene, 1st assembly - reaction C	AATCTGATCNNNNNNCATNNNNNNAGTCGCTGCTAGTTTGCCATCATTCACCGCTTTTTCGCCATCCG GTGTNNNNNNCGCNNNNNNGACCATCACATC
190211 For	*dt016* gene, 1st assembly - reaction C	AGCAGCGACTNNNNNNATGNNNNNNGATCAGATTGGCGCGAAAGGCGTCGAAACCGCAGATAAAGT GCTGAAAGGCGAGAAAGTTCAGGCTAAG
190212 Rev	*dt016* gene, 1st assembly - reaction C	CAAGCTTGCATGCCTGCAGGTCGACTCAGTGGTGGTGGTGGTGGTGGAGCTGCTTAACAACCAGTTT CAGATCAACCGGATACTTAGCCTGAACTTT
190301 For	pSTVPAA before *dt016 gene*, final extension	TGCGAATGCGATGGCAAAAGACACCATCGC
190302 Rev	pSTVPAA after *dt016 gene*, final extension	ACGGCCAGTGCCAAGCTTGCATGCCTGCAGG

*^*a*^NNN indicates positions for introduction of random amino acids.*

### RbsB-Based Bioreporters Assays

RbsB- and DT-based libraries in *E. coli*, or single purified clones were screened for GFPmut2 expression by FACS and/or flow cytometry, as described in detail previously (Tavares et al., 2019). In short, cells were encapsulated and grown to microcolonies in alginate beads, before induction. Beads (microcolonies) expressing higher GFPmut2 signal (measured in the FITC-H channel of the instrument) than the set thresholds were sorted and collected in tubes containing 1 ml LB medium supplemented with Amp and Cm. Sorted mutants were regrown and re-screened either as alginate-bead mixtures or as pure cultures in 96-well plates (in at least eight individually grown replicates). Media, incubation conditions, experiments and instruments details are explained previously (Tavares et al., 2019).

### Statistical Analysis

Flow cytometry induction of GFPmut2 in *E. coli* cultures was measured in multiple independently grown biological replicates (*n* = 6–14). Induction is then expressed as the ratio of the mean GFPmut2 fluorescence of induced cultures by that of their uninduced (split) halves. Differences among the mean GFPmut2 uninduced fluorescence and fold induction were tested using Student’s *t*-test.

### Structure Threading and Analysis

Threaded structures of RbsB variants were determined with Swiss-Model (Guex et al., 2009; Bertoni et al., 2017; Bienert et al., 2017; Waterhouse et al., 2018; Studer et al., 2020) and Phyre2 (Kelley et al., 2015), with wild-type RbsB structure (PDB ID: 2DRI) as scaffold. Structural superpositions and distance analysis were calculated with UCSF Chimera (Pettersen et al., 2004).

## Data Availability Statement

The original contributions presented in the study are included in the article/Supplementary Material, further inquiries can be directed to the corresponding authors.

## Author Contributions

DT carried out all the experiments. DT and JM analyzed data and wrote the main text. Both authors contributed to the article and approved the submitted version.

## Conflict of Interest

The authors declare that the research was conducted in the absence of any commercial or financial relationships that could be construed as a potential conflict of interest.

## Publisher’s Note

All claims expressed in this article are solely those of the authors and do not necessarily represent those of their affiliated organizations, or those of the publisher, the editors and the reviewers. Any product that may be evaluated in this article, or claim that may be made by its manufacturer, is not guaranteed or endorsed by the publisher.
