# Supplementary material for: Ribose-Binding Protein Mutants With Improved Interaction Towards the Non-natural Ligand 1,3-Cyclohexanediol
Source: Front Bioeng Biotechnol. 2021 Jul 23;9:705534. doi: 10.3389/fbioe.2021.705534 (PMC8343135; doi:10.3389/fbioe.2021.705534)
Supplement: Supplementary file 1 [file Data_Sheet_1.docx]

**Supplementary information**

**Ribose-binding protein mutants with improved interaction towards the non-natural ligand 1,3-cyclohexanediol**

Diogo Tavares and Jan Roelof van der Meer

Department of Fundamental Microbiology

University of Lausanne

1015 Lausanne, Switzerland


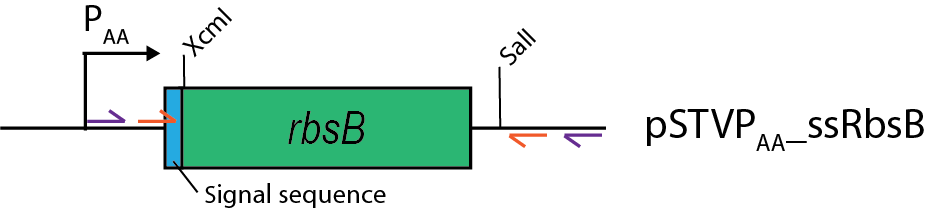


**Figure S1-** Scheme of used primers for DNA shuffling and important restrictions sites. Outer and inner primers are represented in purple and orange, respectively. Not drawn to scale. *rbsB*, indicates coding region for RbsB or the DT mutants.

K D T I A L V V S T L N N P F S V S L K D G A Q K E A D K L G Y N L V V L D S Q N N P A K E L A N V Q D L T V R G T K I L L I V P T D S D A V G N A V K M A N Q A N I P V I T L G S Q A T K G E V V S H I A S D N V L G G K I A G D Y I A K K A G E G A K V I E L Q G I A G A S A A R E R G E G F Q Q A V A A H K F N V L A S Q P A D W D R I K G L N V M Q N L L T A H P D V Q A V F A Q N D E M A L G A L R A L Q T A G K S D V M V V G A D G T P D G E K A V N D G K L A A T I A M L P D Q I G A K G V E T A D K V L K G E K V Q A K Y P V D L K L V V K Q

**Figure S2 –** Amino acid sequence of the DT016 variant. Nine residues mutated based on Rosetta predictions, 1st generation mutations, are highlighted in yellow. Neighboring residues targeted by site saturation mutagenesis are highlighted in blue.
